# *Bougainvillea* Genus: A Review on Phytochemistry, Pharmacology, and Toxicology

**DOI:** 10.1155/2018/9070927

**Published:** 2018-06-24

**Authors:** Rodolfo Abarca-Vargas, Vera L. Petricevich

**Affiliations:** Facultad de Medicina de la Universidad Autónoma del Estado de Morelos (UAEM), Calle Leñeros, Esquina Iztaccíhuatl s/n. Col. Volcanes, Cuernavaca, Morelos, México, C.P. 62350, Mexico

## Abstract

This review discusses the current knowledge of the phytochemistry and* in vitro* and* in vivo* evaluations carried out using the extracts and, where appropriate, the main active components isolated from the genus* Bougainvillea*. Out of 18 species, most phytochemical, pharmacological, and toxicological studies focused on four species with different cultivars and one hybrid. Some plants are used for the treatment of various health disorders. Numerous phytochemical investigations of plants in this genus confirm the presence of aliphatic hydrocarbons, fatty acids, fatty alcohols, volatile compounds, phenolic compounds, peltogynoids, flavonoids, phytosterols, terpenes, carbohydrates, and betalains. Various studies have confirmed that these extracts or active substances that were isolated from the genus* Bougainvillea *have multiple pharmacological activities. Some species of* Bougainvillea* have emerged as sources of traditional medicine in human health. More studies of the phytochemical, pharmacological, and toxicological properties and their mechanisms of action, safety, and efficacy in all* Bougainville*a species, cultivars, and hybrids are advisable for future research.

## 1. Introduction

The genus* Bougainvillea* is a very widespread group throughout the world. It belongs to the family Nyctaginaceae and, according to the “The Plant List”, contains approximately 18 species (*B. berberidifolia, B. buttiana, B. campanulata, B. glabra, B. herzogiana, B. infesta, B. lehmanniana, B*.* lehmannii, B. malmeana, B. modesta, B. pachyphylla, B. peruviana, B. pomacea, B. praecox, B. spectabilis, B. spinosa, B. stipitata, *and* B. trollii*) [1]. Only four species (*B. buttiana, B. glabra, B. spectabilis,* and* B. peruviana*) are commercially exploited [2]. However, there are also more than 100 cultivars and three hybrids, the latter not yet recognized.

The objectives of this review are to provide updated and complete information on the distribution, phytochemical, pharmacological, and toxicity research of* Bougainvillea* species, to identify their therapeutic potential and to direct future research opportunities. The most relevant data were searched using the keyword “*Bougainvillea*” in “Google Scholar”, “PubMed”, “ScienceDirect”, “Scopus”, and “Web of Science”. The taxonomy was validated using the “The Plant List”.

## 2. Ethnobotany

### 2.1. Taxonomical Classification

  Kingdom: Plantae
   Subkingdom: Tracheobionta
     Superdivision: Spermatophyta
      Division: Magnoliophyta
       Class: Magnoliopsida
        Subclass: Caryophyllidae
         Order: Caryophyllales
          Family: Nyctaginaceae
          Genus:* Bougainivillea* [3, 4]

### 2.2. Botanical Characterization and Distribution

The genus* Bougainvillea* is endemic to South America and was firstly reported in Brazil in 1778 before being introduced to Europe, by French military commander Louis Antoine de Bougainville [2]. They are bushes spread in vines or small trees. They also possess stems with internodes and with straight or slightly curved thorns. The leaves are petiolate, elliptical, or wider towards the base. The bracts and flowers are presented in different colours, depending on the species, cultivars, or hybrid. They bloom throughout the year [5].

Botanical textbooks say that some species of the genus* Bougainvillea* are distributed worldwide and without being specific to any single place, species, cultivars, or hybrids. Based on these results, a more exhaustive analysis was performed in the scientific literature. In this review we highlight a more reliable update with respect to the distribution based on the scientific literature or scientific research works.

- ***B. buttiana*:** this species was found in India [6], Mexico [7], and Thailand [8].
- ***B. glabra*:** it was found in Italy [9], Spain, France [10], Bangladesh [11], India [12], China [13], Egypt [14], Israel [15], Thailand [16], Philippines [17], Madagascar [5], Nigeria [18], Hawaii [2], Bolivia, Colombia, Costa Rica, Cuba, Ecuador, El Salvador, United States of America, Guatemala, Honduras, Virgin Islands, Mexico, Nicaragua, Puerto Rico, Dominican Republic, Venezuela [5], and Brazil [19].
- ***B. spectabilis*:** this species was reported in Nigeria [20], Bahamas, Bolivia, Colombia, Costa Rica, Ecuador, Guatemala, Honduras, Jamaica, Mexico, Panama, Puerto Rico, Dominican Republic, Tanzania, Trinidad and Tobago [5], India [21], Montenegro [22], Pakistan [23], Australia, Brazil [19], and Vietnam [24].
- ***B. spinosa*:** this plant species is a very poorly documented plant species, but it was reported in Argentina [25].
- ***B. peruviana*:** this plant is scarce and only noted in the scientific literature in China [13], India [19], and Peru [2].

### 2.3. Bougainvillea Hybrids

Since the beginning of its commercialization, producers have sought to make hybrids of the genus* Bougainvillea* species. They have produced three recognized hybrids with striking characteristics:* B. x spectoperuviana, B. x spectoglabra,* and* B. glabra peruviana* (*B. x buttiana*).

- ***B. x spectoperuviana*:** the first hybrid has no reports in the scientific literature of registration sites or scientific research works according to the databases consulted.
- ***B. x spectoglabra*:** this plant is a hybrid of* B. spectabilis* and* B. peruviana* and it was only reported in China [13].
- ***B. glabraperuviana *or* B. x buttiana*:** this plant is a hybrid of* B. glabra* and* B. peruviana,* and it was identified and reported in Mexico [26], as well as in India, England [19], and China [13]. This plant was thoroughly studied by our working group, and those results are described below.

There is no other current scientific information regarding the location, medical uses, phytochemical profile, or pharmacological or toxicological properties for other species or hybrids.

### 2.4. Synonyms

In the different countries where the* Bougainvillea* are found other popular names were attributed:* Buganvilla* (Spain),* Bugambilia* (Mexico, Guatemala, Cuba, and Philippines),* Pokok bunga kertas *(Malaysia),* Napoleón* (Honduras),* Veranera* (Colombia, Nicaragua, El salvador, Costa Rica, and Panama),* Trinitaria* (Colombia, Panama, Puerto Rico, Dominican Republic, and Venezuela),* Santa Rita* (Argentina, Bolivia, Brazil, Paraguay, and Uruguay),* Primavera, Tres-Marias, Sempre-lustrosa, Santa-rita, Ceboleiro, Roseiro, Roseta, Riso, Pataguinha, Pau-de-roseira, and Flor-de-papel* (Brazil) or* Papelillo* (Northern Peru) [3].

### 2.5. Traditional Medical Use

In traditional medicine the species* B. buttiana*,* B. glabra,* and* B. spectabilis* are indicated for the treatment of coughing [5] and pertussis [7].* B. glabra* is recommended for asthma [27], bronchitis, and dysentery. In a small number of cases, it is indicated for stomach pain, rust, pimples, and blackheads.* B. spectabilis* is also used in other respiratory conditions, including snoring or lung pain, flu, and bronchitis [7]. There are no studies described in the literature regarding the traditional use of the other species and hybrids of* Bougainvillea* in medicine. However, the hybrid* B. x buttiana* was confused with* B. buttiana* since both are distributed and reported in Morelos, Mexico, and both are used to treat cough and whooping cough [5, 28, 29].

## 3. Phytochemistry

The chemical constituents of the genus* Bougainvillea* have been extensively studied since 1970 [9, 37]. The phytochemical analyses were carried out to identify different kinds of components using extracts of different polarities from stems, leaves, or bracts with or without flowers, bark stems, and roots of the species. It has been possible to isolate, identify, and elucidate chemical compounds for species or hybrids. The Marvin program was used to draw the structures of chemical compounds [73].

### 3.1. Aliphatic Hydrocarbons

**Figure 1:**
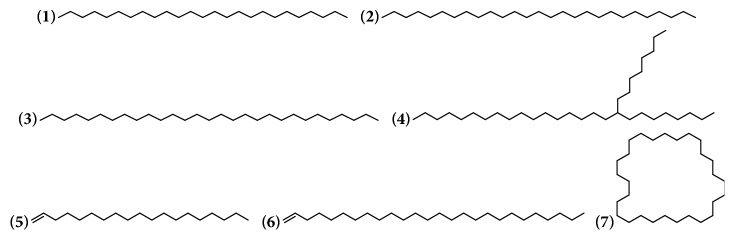
Structure of alkanes, alkenes, and cycloalkanes from genus* Bougainvillea.*

In* Bougainvillea* genus the presence of aliphatic hydrocarbons including alkanes, alkenes, and cycloalkanes has been described. For ethanolic extracts from bracts with flowers from* B. x buttiana* seven of these compounds were found and identified. The presence of this type of compounds,* B*.* x buttiana*, could be considered as an alternative source of energy (Table 1 and Figure 1).

### 3.2. Fatty Acids and Fatty Alcohols

**Figure 2:**
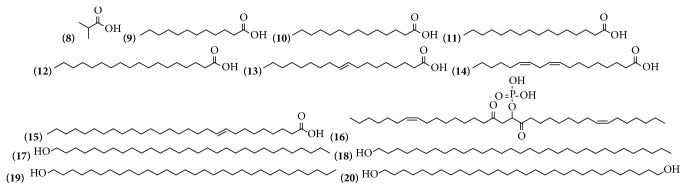
Structure of fatty acid and fatty alcohols from genus* Bougainvillea.*

Fatty acids and fatty alcohols are very common compounds in plants, especially in aerial parts. For the genus of* Bougainvillea,* the presence of 13 of these compounds was verified. Eight compounds were identified in ethanolic extracts from the bracts with flowers from* B. x buttiana* and 5 in ethanolic extracts of the leaves, branches, and roots from* B. spectabilis* (Table 2 and Figure 2).

### 3.3. Volatile Compounds

**Figure 3:**
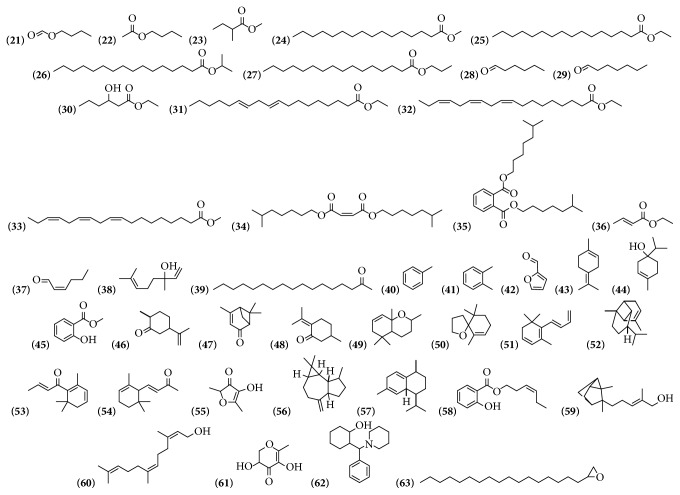
Structure of volatile compounds from genus* Bougainvillea.*

Volatile compounds are compounds that are commonly found in the plant kingdom. Their chemical structures contain some functional groups, including aldehydes, ketones, phenols, oxides, esters, and alcohols. In the leaves and branches of* B. spectabilis* ethanol extract were identified 35 of these compounds. In ethanol, ethanol:water, and ethyl acetate extracts of bracts with flowers of* B. x buttiana* the presence of 9 of these compounds was identified. Only one compound was similar as is the case of the ethyl hexadecanoate compound observed in both extracts* B*.* x buttiana* and* B*.* spectabilis* (Table 3 and Figure 3).

### 3.4. Phenolic Compounds

**Figure 4:**
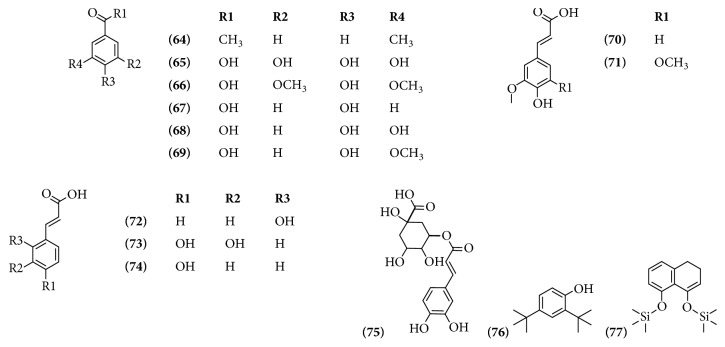
Structure of phenolic compounds from genus* Bougainvillea.*

Phenolic compounds are also widely distributed in the plant kingdom. Fourteen of these compounds have been identified. In the ethanolic extract of bracts with flowers from* B. x buttiana* researchers identified 4 of these compounds. In ethanolic extracts of flowers from* B. glabra* there were 11 phenolic compounds. Compounds** 76** and** 77** are not common in plants; however, their presence is reported in hybrid* B*.* x buttiana* (Table 4 and Figure 4).

### 3.5. Peltogynoids and Flavonoids

**Figure 5:**
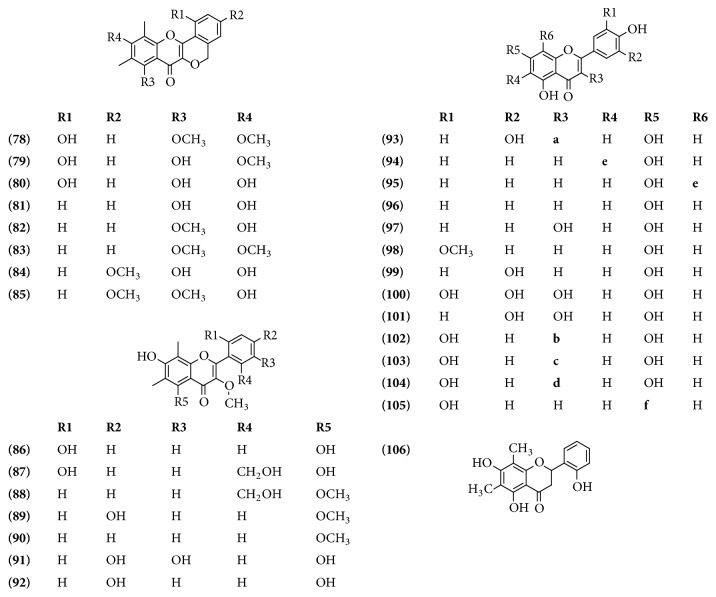
Structure of Peltogynoids and Flavonoids from genus* Bougainvillea*.** a**. Rutinose,** b**. Rhamnopyranoside,** c**. Rhamnopyranosyl-Rhamnopyranosyl-Galactopyranoside,** d**. Caffeoyl-rhamnopyranosyl-Rhamnopyranosyl-Galactopyranoside,** e**. Glucosyl, and** f**. Feruloyl-Apiofuranosyl-Glucopyranoside.

The peltogynoids are restricted in their distribution. In extracts of stem bark from* B. spectabilis* were identified eight peltogynoids. The flavonoids, however, are a group of compounds widely distributed in the plant kingdom, 21 compounds have been identified in* B*.* glabra* and* B*.* spectabilis* (Table 5 and Figure 5).

### 3.6. Phytosterols, Terpenes, and Carbohydrates

**Figure 6:**
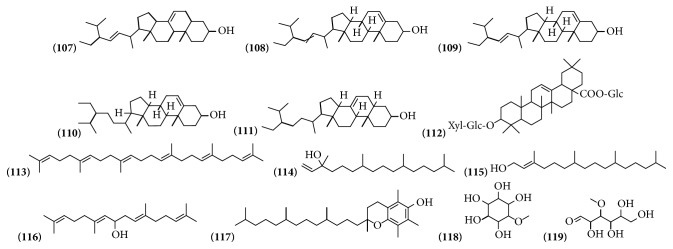
Structure of phytosterols, terpenes, and carbohydrates from genus* Bougainvillea.*

Carbohydrates are chemical compounds that mainly derive from the primary metabolism of vegetables. Sterols and terpenes are secondary metabolites. Out of thirteen compounds identified in the genus* Bougainvillea*, 6 of them were identified from the ethanolic extracts of bracts with flowers from* B. x buttiana*. Four different compounds were identified from extracts of leaves and bracts from* B. glabra*. Three in* B*.* spectabilis* in stem bark, leaves, and branches. Only one compound was similar as is the case of the squalene compound observed in both extracts* B*.* glabra* and* B*.* x buttiana* (Table 6 and Figure 6).

### 3.7. Betalains

**Figure 7:**
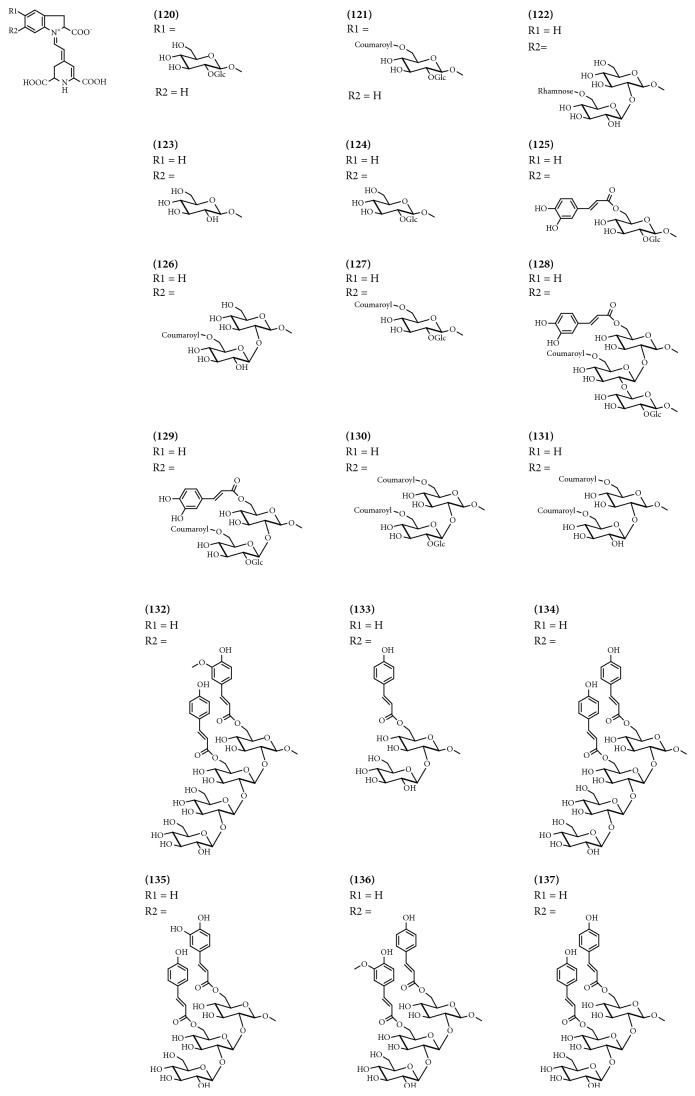
Structure of betalains from genus* Bougainvillea*.

Betalains are vacuolar pigments containing a nitrogenous ring, a ring which is characteristic of the order Caryophyllales. Sixteen of these compounds were identified in bract extracts from the case of extracts of* B. glabra *and 2 were found in extracts from* B. Mrs*. Butt (Table 7 and Figure 7).

## 4. Pharmacological Activity

Four species with different cultivars and one hybrid of* Bougainvillea* have been reported in traditional medicine. A more general view of pharmacological investigations on various crude extracts and isolated chemical compounds of these species is described below.

### 4.1. Analgesic

The analgesic activity was described in two species of* B. glabra* [48] and* B. x buttiana*; in both cases the oral administration was evaluated [29, 49]. For methanol extracts of* B. glabra*, the maximum percentage of analgesia effect obtained using the tail method in male Wistar rats was 79.88% [48]. For the ethanol extracts of* B. x buttiana* (var. Orange), the analgesic effect was studied in female CD1 mice using the acetic acid and formalin methods. For the acetic acid method, the analgesia percentage was 95.65%, while, for formalin method, the extract showed inhibition in both phases [49]. In another study, the analgesic effect of the* B. x buttiana* (var. Rose) ethanol extract was determined after oral administration in BALB/c mice using the acetic acid, tail immersion, and formalin models. For all of the methods used, the extract showed a potent analgesic effect [29].

### 4.2. Anti-Inflammatory

A significant anti-inflammatory activity was obtained in male Wistar rats orally treated with methanol extract of leaves from* B. glabra *[48].

The leaves from* B. spectabilis* were extracted with different solvents, including acetone, alcohol, chloroform, petroleum ether, and chloroform:water. The models used were the oedema induced by carrageenin and the granuloma model induced by cotton pellet, in male Wistar rats. The results indicated that the oral administration in rats with ethanol extract reduced the oedema induced by both methods [50]. In other experiments, the oral administration of methanol extract of leaf from* B*.* spectabilis* was performed and evaluated in Swiss mice using the method of induction oedema with carrageenin and dextran, as well as arthritis induced with Freund's adjuvant in male Wistar albino rats. The results with methanol extract presented an elevated anti-inflammatory activity for all inflammation models [59].

In the case of ethanol extracts of flowers with bracts from* B. x buttiana* (var. Orange), the anti-inflammatory properties were measured using the oedema method induced with carrageenin in female CD1 mice. The amounts of cytokines such as IFN-*γ*, IL-6, and IL-10 and nitric oxide (NO) were also measured. The results obtained from this extract have shown that it is capable of inducing a decrease in TNF productions and an increment in the IL-6, IFN*γ*, IL-10, and NO levels [48]. In another study, the anti-inflammatory effect was obtained in BALB/c female mice orally treated with ethanol extract of bracts and flowers from* B. x buttiana* (var. Rose) [28].

### 4.3. Antipyretic

The oral administration of methanol extracts from* B. glabra* in groups of rats showed a significant antipyretic activity [48].

### 4.4. Antidiabetic

The antidiabetic effects were studied in three species of* Bougainvillea*. The extracts of leaves from* B. glabra* were used in male Wistar rats induced with alloxan [18]. Similar studies were performed with the oral administration of ethanol extracts of flowers from* B. spectabilis*. Its antidiabetic effect was evaluated using diabetic male Wistar rats induced with alloxan [74]. The chloroform extract of flowers from* B. spectabilis* administered intraperitoneally reduced glucose levels in diabetic Swiss mice [75]. The antidiabetic effect was also seen with oral administration of aqueous extract from apical leaves of* B*.* spectabilis *[61, 76]. Studies carried out on the steam bark extract of* B. spectabilis* orally administered in albino rats showed significant hypoglycaemic activity [77]. In the case of ethanol extracts of bracts and flowers from* B*.* x buttiana*, a significant hypoglycaemic activity was observed in female and male CD1 mice orally treated [78].

### 4.5. Antihyperlipidemic

The treatment of diabetic male Wistar rats induced with alloxan or normal Wistar rats orally treated with different extracts from* B. glabra* showed the reduction in the amount of total cholesterol (TC), triglycerides (TG), low-density lipoprotein cholesterol (LDL-Cholesterol), and increase high-density lipoprotein cholesterol (HDL-C) [18]. Another study, using Wistar rats exposed to oral injection of ethanol extract of fresh leaves from* B. spectabilis,* showed a significant reduction in total cholesterol (TC), triglyceride (TG), low-density lipoprotein (LDL), and very low-density lipoprotein (VLDL) levels and significant (*p* <0.01) increase in high-density lipoproteins (HDL) in hypercholesterolemia rats [20].

### 4.6. Antidiarrhoeal

A significant antidiarrhoeal activity was observed in male Wistar rats orally treated with the acetone extract obtained from leaves of* B. glabra* “Choicy” [45].

### 4.7. Antiulcer

The oral administration of extracts of acetone of leaves of* B. glabra* “Choicy” and their antiulcer effect were evaluated in male Wistar rats, and this extract showed a marked antiulcer activity [45].

### 4.8. Antifertility

A reduction in testosterone and oestrogen levels [21] as well as sperm count, viability, and motility [56] was observed in albino Swiss male and female mice orally treated with ethanol extract from* B. spectabilis*.

### 4.9. Neuroprotective

The neuroprotective effect of leaves from* B. glabra* extracted with ethanol was evaluated by the use of the mortality of* Drosophila melanogaster *flies. The results obtained showed that the flies treated with the extract present a lower mortality [32].

The effect of two methanol extracts of* B. spectabilis* from yellow and pink bracts on oxidative stress and neural damage was carried out by use of male Sprague-Dawley rats subcutaneously injected with rotenone. Rotenone provoked significant increases of brain MDA (product of lipid peroxidation) and nitric oxide content along with decreased brain reduced glutathione. There was also a marked and significant inhibition of brain paraoxonase-1 (PON-1) and butyrylcholinesterase (BChE) activities and increased proinflammatory cytokine interleukin-1beta (Il-1*β*) in brain of rotenone-treated rats.* B. spectabilis* flowers extract itself resulted in increased brain oxidative stress, lipid peroxidation, and nitrite content while inhibiting PON-1 activity. The yellow flowers extract inhibited BChE activity and increased brain Il-1*β*. When given to rotenone-treated rats,* B. spectabilis* extracts, however, decreased lipid peroxidation while their low administered doses increased brain glutathione (GSH). Brain nitrite decreased with the pink extract but showed further increase with the yellow extract. Both extracts caused further inhibition of PON-1 activity while the yellow extract resulted in further inhibition of BChE activity. Histopathological studies indicated that both extracts protected against brain, liver, and kidney damage caused by the toxicant [52].

### 4.10. Thrombolytic

The methanol extract of leaves from* B. glabra* [53] and an aqueous extract of green leaves from* B. spectabilis* [54] showed the thrombolytic activity* in vitro* in the blood of healthy volunteers.

### 4.11. Cardiotonic

Cardiotonic evaluation of an aqueous extract of* B. glabra* was performed by using isolated frog heart perfusion technique. The parameters studied included contraction force (HR), heart rate (HR), and cardiac output (CO). This extract provoked an increase in HR and CO [71].

### 4.12. Anthelmintic

For the anthelmintic evaluation, methanol extracts from* B. glabra* were used against species of* Pheretima posthuma *[62], and ethanol* Eudrilus eugeniae* and* Eisenia foetida *[51] were compared with the standards albendazole and metronidazole, respectively. All extracts of* B. glabra* could cause paralysis and death of worms [51, 62].

### 4.13. Antimicrobial

The conventional methods used for the evaluation of biological properties such as antibacterial and antifungal agents of plant extracts include the agar diffusion method and the dilution method [60, 79] (Table 8).

### 4.14. Plant Antiviral

The leaf proteins from* B. x buttiana* were evaluated against RNA viruses such as tobamoviruses, tobacco mosaic virus, and sunnhemp rosette virus. The results obtained showed a degradation of viral RNAs. This implies a great opportunity for control of vegetable viruses [69]. In another study, this purification could identify lysine as the inhibitor of N-glycosidase activity on the 25S rRNA ribosomes of tobacco by interfering with viral multiplication [70].

### 4.15. Cytotoxic

The cytotoxic effect of ethanol extract of leaves from* B. glabra* was evaluated in HT-29 cells, AGS, and BL-13 [16]. Another study with stems and leaves from* B. glabra* extracted with acetonitrile, butanol, dichloromethane, ethyl acetate, hexane, and methanol showed the antiproliferative activity against U373 cells [72].

The effect of the antiproliferative activity in U373 cells was evaluated using extracts hexane, dichloromethane, acetonitrile, ethyl acetate, methanol, and butanol extracts from stems and leaves from* B. spectabilis*. The extract of dichloromethane showed lower antiproliferative activity when compared to others extracts [72]. In another study, the cytotoxic activity of eight new compounds named bougainvinones** 78-85** isolated and elucidated from stem bark was evaluated. The extract from* B. spectabilis* purple from a bipartition was evaluated by using KB, HeLa S-3, HT-29, MCF-7, and HepG2. The results showed that the compound** 84** showed cytotoxicity against cancer cell lines and compounds** 79** and** 80** exhibited cytotoxicity against the KB cell line [24]. In a subsequent study, in ethylacetate extract, five new flavones named bougainvinones** 86**-**90** were isolated and elucidated and their cytotoxic activities were assayed against KB, HepG2, HeLa, S-3, HT-29, and MCF cells. The results showed that all compounds had cytotoxic activity [33].

The cytotoxic effect of flower from* B. x buttiana* extracted in ethanol in bracts of different colours, orange-1 (Bxb01), orange-2 (Bxb02), pink (BxbR), violet (BxbV), and white (BxbW), was studied on HeLa cells. The greater cytotoxic activity was observed with the bracts with flowers Bxb02 and Bxb01 [63]. In another study the evaluation of the cytotoxic activity of different extracts, aqueous, methanol, ethanol, acetone, ethylacetate, dichloromethane, and hexane, of bracts with flowers from* B. x buttiana* was performed. The dichloromethane extract was the most cytotoxic to L-929 cells [26].

### 4.16. Immunomodulatory

The effect of an ethanol extract from* B. x buttiana* on the activation of macrophages of female CD1 mice was determined. The results obtained showed an increase in H_2_O_2_ levels and the extension and formation of vacuoles, reduction of TNF-*α*, and remarkable increases for the levels of IL-10 and NO, suggesting an immunomodulatory effect [55].

### 4.17. Antioxidants

The fresh leaves were degreased with petroleum ether followed by a successive extraction of* B. buttiana* with acetone, ethanol, and distilled water. They were then used for the determination of the antioxidant capacity using the methods of DPPH, FRAP, and inhibition of lipid peroxidation. The ethanol extracts of* B. buttiana* showed antioxidant activity using the two methods studied and an inhibitory activity of lipid peroxidation [63, 64] was observed.

Another study was performed to determine the antioxidant activity of four flowers of a population of Thailand, including* B. glabra*. The extraction was carried out using acidified ethanol and the antioxidant activity was detected using the FRAP and ORAC methods [16]. In another study, the antioxidant activities of different extracts of hexane, dichloromethane, acetonitrile, ethyl acetate, methanol, and butanol were evaluated using the DPPH, ABTS, FRAP, and lipid peroxidation methods. The butanol and methanol extracts showed high antioxidant activity by all methods used [72].* B. glabra* bracts extracted from methanol also showed high levels of NO and antioxidant activity [44, 65–67]. Antioxidant activity was also detected in bracts with flowers extracted in methanol and subsequently partitioned with hexane, chloroform, and water [11].

Fresh leaves from* B. peruviana* were first degreased with petroleum ether and were subsequent to extraction with acetone, distilled water, and ethanol and their antioxidant activity was evaluated by the methods of DPPH and lipid peroxidation inhibition. In ethanolic extracts from* B. peruviana* the antioxidant activity was also detected [64].

Fresh leaves from* B. spectabilis* were extracted with methanol and water. The results showed antioxidant activity in both extracts [68]. The use of apical leaves from* B. spectabilis* extracted with distilled water measured the biomarkers of oxidative stress in blood in diabetic male Wistar rats induced with streptozotocin. The results showed that diabetic rats presented a significant decrease in GSH, SOD, and catalase [61]. Another study of* B. spectabilis* leaves extracted with acetone, chloroform, methanol, petroleum ether, and water also showed the high antioxidant activity [46].

The antioxidant activity of an ethanol extract of bracts of different colours from* B. x buttiana* was determined using the DPPH method. All extracts presented antioxidant activity, and the percent of radical scavenging activity was dependent on the colour of bracts [46]. In another study, the extracts from* B*.* x buttiana* with water, methanol, ethanol, acetone, ethylacetate, dichloromethane, and hexane were used to evaluate their antioxidant activity using the DPPH method. The percent radical scavenging activities in order were methanol > ethanol > water > acetone > ethylacetate > hexane > dichloromethane [26].  Table 9 summarizes the activities found in the genus* Bougainvillea*.

## 5. Toxicity

The leaves of* B. glabra *“Choicy” were subjected to successive extractions with different solvents such as acetone, ethanol, and water. This extract was orally administered in albino rats of both sexes to assess toxicity. The results obtained did not show death in these animals [45].

The evaluation of the toxic effect of aqueous extracts of leaves from* B. spectabilis* was carried out in mice intragastrically treated [7]. Leaves from* B. spectabilis* extracted with ethanol were used in albino male rats (*Rattus norvegicus*) to evaluate the haemoglobin concentration (Hb), packed cell volume (PVC), red blood cell count (RBC), mean corpuscular haemoglobin concentration (MCHC), mean corpuscular volume (MCV), white blood cell count (WBC), and platelet count (PLC). The extract orally administered provoked a significant reduction of Hb, RBC, and PCV [20]. Another investigation with aqueous extracts of leaves from* B. spectabilis* orally administered in male and female albino Swiss mice showed a significant reduction in haemoglobin, red blood cell, and haematocrit levels. This may also be a cause of anemia [80]. The acute toxicity was also evaluated in Wistar albino rats treated with ethanol extracts of root bark. The results showed no toxic effects in the rats [74]. However, the methanol extract from* B. spectabilis* after oral treatment was toxic in male Wistar rats [50]. Other studies showed that the oral administration of methanol extract from* B. spectabilis* in male Swiss mice not was causative of renal or hepatic damage [59].

The toxicity effect of flowers of different colours (white, orange, shocking pink, red, and violet) from* B. spectabilis* extracted with methanol was evaluated on brine shrimp* Artemia salina *[23]. The results obtained showed no toxicity effect presented on* Artemia *[75].

The toxicity effect of ethanol extracts of bracts from* B. x buttiana* was carried out on female CD1 and/or BALB/c mice. The results obtained showed that these extracts were not toxic for two different strains of mice [28].

## 6. Conclusions

This review details the ethnomedical, phytochemical, and pharmacological and toxicological uses of the different species, cultivars, and hybrids of* Bougainvillea*. Although there are several studies on the pharmacological activity of the genus* Bougainvillea*, there is potential of this plant as an anti-inflammatory, antioxidant, immunomodulator, antimicrobial, etc.

## Figures and Tables

**Table 1:** Alkanes, alkenes, and cycloalkanes from genus *Bougainvillea.*

**No.**	**Compound's name**	**Species**	**Parts used**	**Reference**
**(1)**	Pentacosane	*B. x buttiana*	Bracts with flowers	[26]
**(2)**	Heptacosane	*B. x buttiana*	Bracts with flowers	[26]
**(3)**	Nonacosane	*B. x buttiana*	Bracts with flowers	[26]
**(4)**	9-Octylhexacosane	*B. x buttiana*	Bracts with flowers	[26]
**(5)**	1-Nonadecene	*B. x buttiana*	Bracts with flowers	[30]
**(6)**	1-Hexacosene	*B. x buttiana*	Bracts with flowers	[26]
**(7)**	Cyclooctacosane	*B. x buttiana*	Bracts with flowers	[26]

**Table 2:** Fatty acid and fatty alcohols from genus *Bougainvillea.*

**No.**	**Compound's name**	**Species**	**Parts used**	**Reference**
**(8)**	2-Methylpropanoic acid	*B. spectabilis*	Leaves and branches	[22]
**(9)**	Dodecanoic acid	*B. spectabilis*	Leaves and branches	[22]
**(10)**	Tetradecanoic acid	*B. x buttiana*	Bracts with flowers	[26]
**(11)**	Hexadecanoic acid	*B. x buttiana*	Bracts with flowers	[26, 30]
**(12)**	Octadecanoic acid	*B. x buttiana*	Bracts with flowers	[26]
**(13)**	9-Octadecenoic acid (E)-	*B. x buttiana*	Bracts with flowers	[26]
**(14)**	9,12-Octadecadienoic acid (Z,Z)	*B. x buttiana*	Bracts with flowers	[26]
**(15)**	*n*-Octacos-9-enoic acid	*B. spectabilis*	Roots	[31]
**(16)**	1,2-Dipalmitoleoyl glyceryl phosphate	*B. spectabilis*	Roots	[31]
**(17)**	1-Triacontanol	*B. x buttiana*	Bracts with flowers	[26]
**(18)**	1-Dotriacontanol	*B. x buttiana*	Bracts with flowers	[26]
**(19)**	*n*-Hentriacontanol	*B. spectabilis*	Roots	[31]
**(20)**	1,30-Triacontanediol	*B. x buttiana*	Bracts with flowers	[26]

**Table 3:** Volatile compounds from genus *Bougainvillea.*

**No.**	**Compound's name**	**Species**	**Parts used**	**Reference**
**(21)**	Butyl formate	*B. spectabilis*	Leaves and branches	[22]
**(22)**	Butyl acetate	*B. spectabilis*	Leaves and branches	[22]
**(23)**	Methyl 2-methylbutanoate	*B. spectabilis*	Leaves and branches	[22]
**(24)**	Methyl hexadecanoate	*B. spectabilis*	Leaves and branches	[22]
**(25)**	Ethyl hexadecanoate	*B. spectabilis*	Leaves and branches	[22]
		*B. x buttiana*	Bracts with flowers	[26]
**(26)**	Isopropyl palmitate	*B. x buttiana*	Bracts with flowers	[30]
**(27)**	Propyl hexadecanoate	*B. spectabilis*	Leaves and branches	[22]
**(28)**	Hexanal	*B. spectabilis*	Leaves and branches	[22]
**(29)**	Heptanal	*B. spectabilis*	Leaves and branches	[22]
**(30)**	Ethyl 3-hydroxy-hexanoate	*B. spectabilis*	Leaves and branches	[22]
**(31)**	9,12-Octadecadienoic acid, ethyl ester	*B. x buttiana*	Bracts with flowers	[26]
**(32)**	9,12,15-Octadecatrienoic acid, ethyl ester, (Z,Z,Z)	*B. x buttiana*	Bracts with flowers	[26]
**(33)**	Methyl linolenate	*B. spectabilis*	Leaves and branches	[22]
**(34)**	Diisooctyl maleate	*B. x buttiana*	Bracts with flowers	[30]
**(35)**	1,2-Benzenedicarboxylic acid, diisooctyl ester	*B. x buttiana*	Bracts with flowers	[30]
**(36)**	Ethyl (E)-crotonate	*B. spectabilis*	Leaves and branches	[22]
**(37)**	(Z)-2-Hexenal	*B. spectabilis*	Leaves and branches	[22]
**(38)**	Linaool	*B. spectabilis*	Leaves and branches	[22]
**(39)**	2-Heptadecanone	*B. spectabilis*	Leaves and branches	[22]
**(40)**	Toluene	*B. spectabilis*	Leaves and branches	[22]
**(41)**	*O*-xylene	*B. spectabilis*	Leaves and branches	[22]
**(42)**	2-Furfural	*B. spectabilis*	Leaves and branches	[22]
**(43)**	Terpinolene	*B. spectabilis*	Leaves and branches	[22]
**(44)**	Terpinen-4-ol	*B. spectabilis*	Leaves and branches	[22]
**(45)**	Methyl salicylate	*B. spectabilis*	Leaves and branches	[22]
**(46)**	Trans-dihydrocarvone	*B. spectabilis*	Leaves and branches	[22]
**(47)**	Verbenone	*B. spectabilis*	Leaves and branches	[22]
**(48)**	Pulegone	*B. spectabilis*	Leaves and branches	[22]
**(49)**	Dihydroedulan II	*B. spectabilis*	Leaves and branches	[22]
**(50)**	Theaspirane B	*B. spectabilis*	Leaves and branches	[22]
**(51)**	Dehydroionene	*B. spectabilis*	Leaves and branches	[22]
**(52)**	*α*-copaene	*B. spectabilis*	Leaves and branches	[22]
**(53)**	(E)-*β*-damascenone	*B. spectabilis*	Leaves and branches	[22]
**(54)**	*α*-(E)-Ionone	*B. spectabilis*	Leaves and branches	[22]
**(55)**	2,5-Dimethyl-4-hydroxy-3(2H)-furanone	*B. spectabilis*	Leaves and branches	[22]
**(56)**	Aromadendrene	*B. spectabilis*	Leaves and branches	[22]
**(57)**	Cadina-1.4-diene	*B. spectabilis*	Leaves and branches	[22]
**(58)**	(Z)-3-Hexenyl salicylate	*B. spectabilis*	Leaves and branches	[22]
**(59)**	*α*-santalol	*B. spectabilis*	Leaves and branches	[22]
**(60)**	(Z,Z)-farnesol	*B. spectabilis*	Leaves and branches	[22]
**(61)**	4H-Pyran-4-one, 2,3-dihydro-3,5-dihydroxy-6-methyl	*B. x buttiana*	Bracts with flowers	[26]
**(62)**	2-(Phenyl-piperidin-1-yl-methyl)-cyclohexanol	*B. x buttiana*	Bracts with flowers	[26]
**(63)**	Oxirane, heptadecyl-	*B. x buttiana*	Bracts flowers	[26]

**Table 4:** Phenolic compounds from genus *Bougainvillea.*

**No.**	**Compound's name**	**Species**	**Parts used**	**Reference**
**(64)**	Ethanone, 1-(2-hydroxy-5-methylphenyl)	*B. x buttiana*	Bracts with flowers	[26]
**(65)**	Gallic acid	*B. glabra*	Flowers	[16]
**(66)**	Syringic acid	*B. glabra*	Flowers	[16]
**(67)**	*p*-Hydroxybenzoic acid	*B. glabra*	Flowers	[16]
**(68)**	Protocatechuic acid	*B. glabra*	Flowers	[16]
**(69)**	Vanillic acid	*B. glabra*	Leaves	[32]
**(70)**	Ferulic acid	*B. glabra*	Flowers	[16]
**(71)**	Sinapic acid	*B. glabra*	Flowers	[16]
**(72)**	2-Propenoic acid, 3-(2-hydrophenyl)-(E)-	*B. glabra*	Flowers	[16]
		*B. x buttiana*	Bracts with flowers	[30]
**(73)**	Caffeic acid	*B. glabra*	Flowers	[16]
**(74)**	Coumaric acid	*B. glabra*	Leaves	[32]
**(75)**	Chlorogenic acid	*B. glabra*	Flowers	[16]
**(76)**	2,4-Di-tert-butylphenol	*B. x buttiana*	Bracts with flowers	[26]
**(77)**	Naphthalene, 3,4-dihydro-1,8-bis (trimethylsilyloxy)	*B. x buttiana*	Bracts with flowers	[26]

**Table 5:** Peltogynoids and flavonoids from genus *Bougainvillea.*

**No.**	**Compound's name**	**Species**	**Parts used**	**Reference**
**(78)**	Bougainvinone A	*B. spectabilis*	Stem bark	[24]
**(79)**	Bougainvinone B	*B. spectabilis*	Stem bark	[24]
**(80)**	Bougainvinone C	*B. spectabilis*	Stem bark	[24]
**(81)**	Bougainvinone D	*B. spectabilis*	Stem bark	[24]
**(82)**	Bougainvinone E	*B. spectabilis*	Stem bark	[24]
**(83)**	Bougainvinone F	*B. spectabilis*	Stem bark	[24]
**(84)**	Bougainvinone G	*B. spectabilis*	Stem bark	[24]
**(85)**	Bougainvinone H	*B. spectabilis*	Stem bark	[24]
**(86)**	Bougainvinone I	*B. spectabilis*	Stem bark	[33]
**(87)**	Bougainvinone J	*B. spectabilis*	Stem bark	[33]
**(88)**	Bougainvinone K	*B. spectabilis*	Stem bark	[33]
**(89)**	Bougainvinone L	*B. spectabilis*	Stem bark	[33]
**(90)**	Bougainvinone M	*B. spectabilis*	Stem bark	[33]
**(91)**	5,7,3′,4′-Tetrahydroxy-3-methoxy-6,8-dimethylflavone	*B. spectabilis*	Stem bark	[33]
**(92)**	5,7,4′-Trihydroxy-3-methoxy-6,8-dimethylflavone	*B. spectabilis*	Stem bark	[33]
**(93)**	Rutin	*B. glabra*	Flowers	[16]
**(94)**	Isovitexin	*B. glabra*	Leaves	[14]
**(95)**	Vitexin	*B. glabra*	Leaves	[14]
**(96)**	Apigenin	*B. glabra*	Flowers	[16]
**(97)**	Kaempferol	*B. glabra*	Flowers	[16]
**(98)**	Chrysoeriol	*B. glabra*	Leaves	[14]
**(99)**	Luteolin	*B. glabra*	Leaves	[14]
**(100)**	Myricetin	*B. glabra*	Flowers	[16]
**(101)**	Quercetin	*B. glabra*	Flowers	[16]
**(102)**	Quercitrin	*B. glabra*	Stem bark	[34]
**(103)**	Quercetin 3-*O*-*α*-L-(rhamnopyranosyl)(1→6)-[*α*-L-rhamnopyranosyl(1→2)]-*β*-D-galactopyranoside	*B. glabra*	Bracts	[35]
**(104)**	Quercetin 3-*O*-*α*-L-(4-caffeoylrhamnopyranosyl)(1→6)-[*α*-L-rhamnopyranosyl(1→2)]-*β*-D-galactopyranoside	*B. glabra*	Bracts	[35]
**(105)**	Luteolin-7-*O*-[2′′-*O*-(5′′′-*O*-feruloyl)-*β*-D-apiofuranosyl]-*β*-D-glucopyranoside	*B. glabra*	Leaves	[14]
**(106)**	2′-Hydroxydemethoxymatteucinol	*B. spectabilis*	Stem bark	[33]

**Table 6:** Phytosterol, terpenes, and carbohydrates from genus *Bougainvillea.*

**No.**	**Compound's name**	**Species**	**Parts used**	**Reference**
**(107)**	Chondrillasterol	*B. x buttiana*	Bracts with flowers	[26]
**(108)**	Stigmasta-5,22-dien-3-ol	*B. x buttiana*	Bracts with flowers	[26]
**(109)**	Stigmasterol	*B. glabra*	Leaves	[32]
**(110)**	*β*-sitosterol	*B. spectabilis*	Stem bark	[34]
**(111)**	Stigmast-7-en-3-ol, (3*β*,5*α*)	*B. x buttiana*	Bracts with flowers	[26]
**(112)**	Momordin IIc	*B. glabra*	Bracts	[35]
**(113)**	Squalene	*B. glabra*	Leaves	[32]
		*B. x buttiana*	Bracts with flowers	[32]
**(114)**	Isophytol	*B. spectabilis*	Leaves and branches	[22]
**(115)**	Phytol	*B. spectabilis*	Leaves and branches	[22]
**(116)**	Geranylgeraniol	*B. glabra*	Leaves	[32]
**(117)**	*α*-Tocopherol	*B. x buttiana*	Bracts with flowers	[26]
**(118)**	Pinitol	*B. spectabilis*	Leaves	[36]
**(119)**	3-O-Methyl-D-glucose	*B. x buttiana*	Bracts with flowers	[26]

**Table 7:** Betalains from genus *Bougainvillea.*

**No.**	**Compound's name**	**Species**	**Parts used**	**Reference**
**(120)**	Bougainvillein-r-I	*B.* Mrs. Butt	Bracts	[37]
**(121)**	Bougainvillein-r-III	*B.* Mrs. Butt	Bracts	[37]
**(122)**	15S-Betanidin 6-*O*-*β*-glucoside	*B. glabra*	Bracts	[38]
**(123)**	6′′-O-Rhamnosyl-bougainvillein-V	*B. glabra*	Bracts	[39]
**(124)**	Bougainvillein-V	*B. glabra*	Bracts	[38]
**(125)**	15S-Betanidin 6-*O*(6′-*O*-E-caffeoyl)-*β*-sophoroside	*B. glabra*	Bracts	[38]
**(126)**	15S-Betanidin 6-*O*(6′′-*O*-E-4-coumaroyl)-*β*-sophoroside	*B. glabra*	Bracts	[38]
**(127)**	15S-Betanidin 6-*O*(6′-*O*-E-4-coumaroyl)-*β*-sophoroside	*B. glabra*	Bracts	[38]
**(128)**	15S-Betanidin 6-*O*{2′′-*O*-*β*-sophorosyl[(6′-*O*-E-caffeoyl)-(6′′-*O*-E-4-coumaroyl)}-*β*-sophoroside	*B. glabra*	Bracts	[38]
**(129)**	15S-Betanidin 6-*O*-{2′′-*O*-*β*-glucosyl)[6′-*O*-E-caffeoyl)-(6′′-*O*-E-4-coumaroyl)]}-*β*-sophoroside	*B. glabra*	Bracts	[38]
**(130)**	15S-Betanidin 6-*O*-[2′′-*O*-*β*-glucosyl)(6′,6′′-di-4-coumaroyl)]-*β*-sophoroside	*B. glabra*	Bracts	[38]
**(131)**	15S-Betanidin 6-*O*(6′,6′′-di-*O*-E-4-coumaroyl)-*β*-sophoroside	*B. glabra*	Bracts	[38]
**(132)**	Betanidin-6-O-[(2′′-O-beta-sophorosyl)-(6′-O-trans-feruloyl-6′′-O-trans-coumaroyl)]-beta-sophoroside	*B. glabra*	Bracts	[40]
**(133)**	Betanidin-6-O-(6′-O-trans-4-coumaroyl)-*β*-sophoroside	*B. glabra*	Bracts	[40]
**(134)**	Betanidin-6-O-[(2′′-O-*β*-sophorosyl)-(6′,6′′-di-O-trans-coumaroyl)]-*β*-sophoroside	*B. glabra*	Bracts	[40]
**(135)**	Betanidin-6-O-[(2′′-O-*β*-glucosyl)-(6′-O-trans-caffeoyl-6′′-O- trans-coumaroyl)]-*β*-sophoroside	*B. glabra*	Bracts	[40]
**(136)**	Betanidin-6-O-[(2′′-O-*β*-glucosyl)-(6′-O-trans-coumaroyl-6′′-O-trans-feruloyl)]-*β*-sophoroside	*B. glabra*	Bracts	[40]
**(137)**	Betanidin-6-O-[(2-O-*β*-glucosyl)-(6′,6′′-di-O-trans-coumaroyl)]-*β*-sophoroside	*B. glabra*	Bracts	[40]

**Table 8:** Antibacterial and antifungal activities found in genus *Bougainvillea.*

**Microorganism**	**Species**	**Extract**	**Reference**
*Bacillus subtilis*	*B. spectabilis*	Methanol	[23]
	*B. spectabilis*	Ethanol, water, chloroform, and ethyl acetate	[41]
	*B. spectabilis*	Chloroform partition	[42]
	*Snow White*	Ethanol	[43]
	*B. glabra*	Methanol	[11, 44]
	*B. glabra* “Choicy”	Acetone	[45]
*Bacillus megaterium*	*B. spectabilis*	Chloroform partition	[42]
*Bacillus cereus*	*B. spectabilis*	Chloroform partition	[42]
*Escherichia coli*	*B. spectabilis*	Methanol	[23]
	*B. spectabilis*	Chloroform partition	[42]
	*B. spectabilis*	Acetone, chloroform, methanol, petroleum ether, and water	[46]
	*B. glabra* “Choicy”	Acetone	[45]
	*Snow White*	Ethanol	[43]
	*B. glabra*	Methanol	[11, 44]
*Shigella flexneri*	*B. spectabilis*	Methanol	[23]
*Shigella boydii*	*B. spectabilis*	Chloroform partition	[42]
*Shigella dysenteriae*	*B. spectabilis*	Chloroform partition	[42]
*Sarcina lutea*	*B. spectabilis*	Chloroform partition	[42]
*Klebsiella pneumoniae*	*B. spectabilis*	Aqueous	[41]
	*B. spectabilis*	Acetone, chloroform, methanol, petroleum ether, and water	[46]
	*B. glabra* “Choicy”	Acetone	[45]
	*Snow White*	Ethanol	[43]
*Staphylococcus aureus*	*B. spectabilis*	Chloroform partition	[42]
	*B. spectabilis*	Petroleum ether and chloroform	[46]
	*B. glabra* “Choicy”	Acetone	[45]
	*Snow White*	Ethanol	[43]
*Proteus vulgaris*	*B. spectabilis*	Ethanol and chloroform	[41]
	*B. glabra* “Choicy”	Acetone	[45]
	*Snow White*	Ethanol	[43]
*Salmonella typhi*	*B. spectabilis*	Methanol	[23]
	*B. spectabilis*	Chloroform partition	[42]
	*Snow White*	Ethanol	[43]
*Salmonella paratyphi*	*B. spectabilis*	Chloroform partition	[42]
*Vibrio cholerae*	*B. spectabilis*	Acetone, chloroform, methanol, and petroleum ether	[46]
	*Snow White*	Ethanol	[43]
*Vibrio mimicus*	*B. spectabilis*	Chloroform partition	[42]
*Vibrio parahaemolyticus*	*B. spectabilis*	Chloroform partition	[42]
*Pseudomonas aeruginosa*	*B. spectabilis*	Methanol	[23]
	*B. spectabilis*	Chloroform partition	[42]
	*B. glabra*	Methanol	[11, 44]
*Candida albicans*	*B. spectabilis*	Chloroform partition	[42]
	*B. glabra*	Methanol	[47]
*Aspergillus fumigatus*	*B. spectabilis*	Acetone, chloroform, methanol, petroleum ether, and water	[46]
	*B. glabra*	Methanol	[47]
*Aspergillus flavus*	*B. spectabilis*	Acetone, chloroform, methanol, petroleum ether, and water	[46]
*Aspergillus niger*	*B. spectabilis*	Ethanol, water, and chloroform	[41]
	*B. spectabilis*	Chloroform partition	[42]
	*B. spectabilis*	Acetone, chloroform, methanol, petroleum ether, and water	[46]
*Trichoderma viridae*	*B. spectabilis*	Ethanol and chloroform	[41]
*Penicillium notatum*	*B. spectabilis*	Ethanol	[41]
*Rhizopus oryzae*	*B. spectabilis*	Ethyl acetate	[41]
*Coccidioides immitis*	*B. glabra*	Methanol	[47]

**Table 9:** Summarized activities found in genus *Bougainvillea.*

**Activity**	**Species**					**References**
	***B. buttiana***	***B. glabra***	***B. spectabilis***	***B. peruviana***	***B. x buttiana***	
Analgesic		√			√	[29, 48, 49]
Anthelmintic		√				[50, 51]
Antidiabetic		√	√		√	[16, 42, 52–55]
Antidiarrheal		√				[45]
Antifertility			√			[21, 56]
Antihyperlipidemic		√	√			[18, 29, 57, 58]
Anti-inflammatory		√	√		√	[28, 48–50, 59]
Antimicrobial		√	√			[11, 23, 41–47, 60]
Antioxidants	√	√	√	√	√	[11, 16, 26, 46, 61–68]
Antipyretic		√				[48]
Antiulcer		√				[45]
Antiviral					√	[69, 70]
Cardiotonic		√				[71]
Cytotoxic		√			√	[16, 24, 26, 33, 63, 72]
Immunomodulator					√	[55]
Neuroprotective		√	√			[32, 52]
Thrombolytic		√	√			[53, 54]

**Note:** the symbol √ represents the present biological or pharmacological activity in the respective species.
